# Probation clients’ barriers to access and use of opioid use disorder medications

**DOI:** 10.1186/s40352-019-0089-6

**Published:** 2019-05-28

**Authors:** Jessica Reichert, Lily Gleicher

**Affiliations:** Illinois Criminal Justice Information Authority, Center for Justice Research and Evaluation, 300 W. Adams St., Suite 200, Chicago, IL 60606 USA

**Keywords:** Medication-assisted treatment, MAT, Methadone, Buprenorphine, Naltrexone, Probation, Opioid, Substance use

## Abstract

**Background:**

There is a gap between evidence-based treatment with medications for opioid use disorders (OUDs) and current practices of probation departments who supervise individuals with OUDs. Many probationers with OUDs cannot access FDA-approved medications to treat their disorders despite the strong evidence of their effectiveness. The barriers to medications for those under probation supervision include practitioners’ negative attitudes toward medications, costs, stigma, and diversion risk. Probation officers have an ethical obligation to help their clients reduce barriers to access the care they need which in turn can improve their outcomes and increase public safety.

**Results:**

The current study explores how probation departments respond to probationers with OUDs, focusing on the barriers to accessing OUD medications based on a survey of probation department directors/administrators (hereafter referred to as probation department leaders) in Illinois (*N* = 26). A majority of probation department leaders reported perceived staff barriers to their clients accessing medications. Reasons included lack of medical personnel experience, cost, need for guidance on medications, and regulations set by their organization or jurisdiction that prohibit client use of medications. Probation department leaders reported knowing less about the use of methadone and how it is administered, compared to buprenorphine and naltrexone. In addition, probation department leaders were generally more open to referring clients for treatment that include buprenorphine or naltrexone compared to methadone. Despite slightly less training or familiarity with methadone than the other medications, the number of probation department leaders who knew where to refer someone for each of the three FDA-approved medications was similar.

**Conclusions:**

The current study found probation department leaders perceive some barriers to their staff linking or referring their clients to OUD medications. Study findings indicate a need for administration- and staff-level training, interagency collaboration, and policy changes to increase access to, education on, and use of, medications for probation clients. Such efforts will ultimately help probation clients with OUDs stabilize and adhere to other probation requirements and engage in behavioral therapy, which may result in positive outcomes such as reduced recidivism, increased quality of life, and reduced mortality.

## Background

Probation departments are charged with management of probation clients for public safety and to aid in rehabilitation, which includes helping clients with opioid use disorders (OUDs) gain access to the treatment and recovery services they need (Lovins, Cullen, Latessa, & Lero Jonson, 2018). Despite the availability of effective FDA-approved medications to treat OUDs, many probationers cannot access them (Legal Action Center, 2011; Mitchell et al., 2016). This may be due in part to probation department restrictions, as well as other individual or community factors that limit accessibility (Mitchell et al., 2016). There is an apparent gap between evidence-based treatment with medications for OUD and current practices of U.S. probation departments (Ducharme, Chandler, & Wilery, 2013).

At year-end of 2016, there were 3.7 million people on probation, many of whom are on probation for drug-related offending (Fletcher, 2014; Kaeble, 2018; Lovins et al., 2018). Due to limited data availability, it is difficult to know the precise prevalence of substance use disorders (SUDs), including OUDs, of U.S. probationers (Fearn et al., 2016; Kaeble, 2018). However, it is estimated between 60% to 80% of individuals supervised in the community (probation and parole) have a substance-related issue, which is higher than the general population (Feucht & Gfroerer, 2011). A national study revealed SUD prevalence rates of probationers and parolees were between four to nine times higher than non-probationers (Fearn et al., 2016). In a national survey of drug courts, respondents noted the prevalence of probationers with a SUD whose primary substance was opioids was 34% in suburban drug courts, 31% in rural drug courts, and 22% in urban drug courts (Marlow, Hardin, & Fox, 2016).

Those on probation must comply with requirements—or conditions—of their sentencing order, as well as any additional requirements dictated by their probation officer, to successfully complete probation supervision. For those with an OUD, these conditions may include outpatient or inpatient SUD treatment; drug testing; linkage to other social and human service agencies; and/or attending recovery support groups, such as narcotics anonymous (NA) or SMART recovery groups (Gryczynski et al., 2012). Although SUDs are chronic, relapsing conditions, probationers who fail to remain drug-free may face sanctions, which can include probation revocation or incarceration (Richmond, 2018).

### SUD treatment in probation

Currently, the criminal justice system is the main referral method to SUD treatment—nearly half of all treatment referrals (Farabee, et al., 1998; Substance Abuse and Mental Health Services Administration [SAMHSA], 2014). While probation officers do not have control over the quality or evidence-base of services provided to their clients by treatment providers, a probation officer’s role as a behavioral change agent includes linking their clients to appropriate services that help target their criminogenic needs—or those needs that are associated with potential recidivism—which includes SUDs (Andrews & Bonta, 2010).

More frequently, this linkage to SUD treatment services may incorporate evidence-based (e.g. cognitive-behavioral therapy, dialectical behavioral therapy, moral reconation therapy, contingency management) and/or non-evidence-based/less effective treatment approaches (e.g. drug education, twelve step/traditional approaches, unstructured and vague rehabilitation programs) for SUDs (Andrews & Bonta, 2010; Latessa, Listwan, & Koetzle, 2014; Van Voorhis & Salisbury, 2014). Findings from the National Criminal Justice Treatment Program (NCJTP) survey found that fewer than 60% of specified evidence-based practices were used in treatment programs offered to drug-involved offenders among 384 criminal justice and community-based programs (including 191 probation or parole programs and 191 community agencies) in the U.S. (Friedmann, Taxman, & Henderson, 2006). In addition, findings from this survey also indicate the most frequently provided treatment services are drug and alcohol education and less than four hours a week of outpatient counseling, which may be insufficient based on the depth of an offender’s SUD (Belenko & Peugh, 2005; Taxman, Perdoni, & Harrison, 2007). In their study on MAT in corrections, Friedmann and the MAT Working Group of Criminal Justice-Drug Abuse Treatment Studies (CJ-DATS) found that probation and parole agencies were least likely to link individuals to MAT for either medication maintenance or medical detoxification (Friedmann et al., 2012).

Research from the past several decades shows evidence that supports the efficacy of treatment programs that adhere to the following (Andrews & Bonta, 2010; Bahr, Masters, & Taylor, 2012; Cullen & Gendreau, 2000; Friedmann, Taxman, & Henderson, 2006; Lipsey, 1999; Simpson, 2004):

1. Use of appropriate standardized assessments and treatment matching.
2. Use behavioral therapies, including motivational techniques to help with offender motivation to change.
3. Target criminogenic factors based on a validated risk/needs assessment.
4. Are responsive to individual styles of learning and potential barriers to engaging in treatment (e.g. transportation, mental health, language, intellectual/cognitive functioning).
5. Use well-trained professionals.
6. Maintain fidelity to the core program or practice components (what make it evidence-based) and core correctional practices.

While important supplementary services, self-help groups and peer recovery support groups (e.g. Alcoholics Anonymous, Al-Anon, Narcotics Anonymous, and other 12-step based programs)—the most frequently offered SUD service—are insufficient as the only form of service provided to individuals with SUDs (Van Voorhis & Salisbury, 2014). Further, most offenders have more than one criminogenic need, in addition to substance use, that may be a contributing factor of their risk to recidivate (Latessa et al., 2014). More generally, other criminogenic needs may likely be intertwined with or contribute to substance using behavior, such as antisocial attitudes, values, and beliefs (i.e. “I’m not hurting anyone by using”); antisocial personality traits (e.g. impulsivity, poor coping skills); and/or peer associations (Andrews & Bonta, 2010; Latessa et al., 2014).

Matching assessments to treatment needs requires the consideration of the medical aspect of SUDs—particularly OUDs. The linkage made by probation officers also should include referral to medical practitioners for assessment for the potential use of effective, evidence-based medications (Krawczyk, Picher, Feder, & Saloner, 2017; U.S. Food & Drug Administration, 2017). However, only 5% of those referred by the criminal justice system to OUD treatment receive agonist treatment (methadone or buprenorphine), compared to 41% of persons referred by other sources (Krawczyk et al., 2017). Currently, three medications are FDA-approved to treat OUD—methadone, buprenorphine, and injectable naltrexone. When medication is combined with behavioral therapy, it is referred to as medication-assisted treatment (MAT) (Reichert, Gleicher, & Salisbury-Afshar, 2017; Substance Abuse and Mental Health Services Administration, 2014); however, use of methadone or buprenorphine sans behavioral therapy has also been shown to be effective (particularly with such lengthy wait lists for behavioral therapy) when compared to counseling alone (Mattick, Breen, Kimber, & Davoli, 2009; Weiss et al., 2011). This, in turn, has resulted in increased expansion of drug-involved offenders in corrections due to the prevalence of criminal justice sanctions in lieu of effective therapeutic approaches such as MAT (Friedmann et al., 2012).

Methadone and buprenorphine, which are full and partial agonists respectively, have substantial research supporting their efficacy with OUDs, including decreased opioid-related mortality (Fullerton et al., 2014; Gibson et al., 2008; Schwartz et al., 2013), improved treatment retention (Connock et al., 2007; Mattick et al., 2009; Thomas et al., 2014), reduced subsequent drug use (Perry et al., 2013; Thomas et al., 2014), reduced HIV risk behavior (Meltzer et al., 2011), and reduced recidivism (Fullerton et al., 2014; Perry et al., 2013). Injectable naltrexone has been shown to decrease rate of relapse (over 24-weeks), increase time to relapse, and reduce subsequent drug use (Lee et al., 2015); however, less is known about the long-term use of injectable naltrexone. Retention could be improved for all OUD medications, but continued use and retention of individuals using methadone or buprenorphine is higher than continued use of injectable naltrexone (Bart, 2012; Kjome & Moeller, 2011; Morgan, Schackman, Leff, Linas, & Walley, 2018; Soyka, Singg, Koller, & Kuefner, 2008); however, to date, most research has compared the use of methadone and buprenorphine, whereas less research is available on the comparison of all three medications and the comparison of naltrexone to buprenorphine or methadone.

Prior research has noted barriers to accessing OUD medications for criminal justice populations (Ducharme et al., 2013; Farabee, 2018; Friedmann et al., 2012; Rich et al., 2015). Limited access of justice-involved individuals to effective medications may also set some probationers up for potential failure who may otherwise benefit from these medications. In part, inadequate access of justice-involved individuals to OUD medications may be due to a limited number of treatment providers who offer long-term, individualized medication-assisted treatment to their clients (not medical detox or required taper). While the number of clients treated with methadone has increased over the past several years—227,003 in 2003 to 356,843 in 2015—the number of treatment facilities with opioid treatment programs (OTPs) rose slightly, though remained low, from 8 % in 2003 to 10% in 2015 (Alderks, 2017). Among OTPs, the number offering buprenorphine rose from 11% in 2013 to 58% in 2015; the number of non-OTP treatment facilities offering buprenorphine increased from 5 % in 2003 to 21% in 2015 (Alderks, 2017). Eleven percent of OTPs and 8 % of non-OTP facilities provided naltrexone in 2011, increasing in 2015 to 23% and 16%, respectively (Alderks, 2017).

Other factors that may influence correctional agencies’ limited use or linkage to MAT, include: a preference for abstinence-based treatment without OUD medications; concerns about liability issues; belief that MAT is offered by community treatment programs; lack of qualified staff; lack of knowledge regarding the clinical efficacy and use of MAT for criminal justice populations; treatment philosophy of the agency; regulations or mandates from the judge/court; and staff objections to the use of MAT including stigmatizing (Ducharme et al., 2013; Farabee, 2018; Friedmann et al., 2012; Mitchell et al., 2016). In addition, probation officers may not know where or how to link their clients to OTPs, buprenorphine prescribers, or other health or treatment facilities that provide these services. Per the Medication Assisted Treatment Implementation in Community Correctional Environments (MATICCE) study, a multi-site cluster randomized study analyzing two implementation strategies to assist in increasing offender access and referrals to MAT available in the community, found officers who had more knowledge about MAT, who viewed their role as assisting clients’ community reintegration, and who identified the importance of substance use disorder treatment to meet those needs generally held more favorable views of methadone or buprenorphine (Mitchell et al., 2016). Further, prominent among the 118 semi-structured interviews was limited understanding of substance use disorders and/or MAT benefits, viewing MAT as useful for detoxification or short-term stabilization, but did not support MAT for maintenance medication (Mitchell et al., 2016).

Probation department leaders and officers should work to reduce barriers and provide access and information on each potentially life-saving medication, offering probationers various avenues for probation success and increased quality of life (Ducharme et al., 2013). In fact, it is argued, that probation officers have an ethical obligation to help ensure that their clients access to the care they need (Bruce & Schleifer, 2008), especially seeing as access to these medications can help rehabilitate drug-involved offenders, reduce risk for mortality and morbidity, while also keeping the community safe.

Despite probation’s role in assisting individuals to obtain treatment and services related to criminogenic needs—including substance misuse and SUDs—there is still only minimal information about what barriers probation departments face when assisting their clients. This study addresses this gap in knowledge. Because evidence-based treatment incorporates an individualized treatment plan, probation clients with OUD should have information and the ability to access all medication options available to them, as deemed appropriate by a medical professional—not a judge, probation officer, or other correctional staff. The current study adds to the research and further explored the perspectives of probation department directors and administrators on how they perceive their agency responds to probationers with OUDs, focusing on the barriers to accessing medications, as well as probation departments’ familiarity with, and training on, each of the three FDA-approved OUD medications.

## Methods/design

Researchers conducted an online survey of Illinois probation department leaders. The survey consisted of 32 questions derived from the CJ-DATS 2 Survey: Opinions about MAT, perceptions about pharmacotherapy for opioid dependence (Taxman, Young, Wiersema, Rhodes, & Mitchell, 2007). An additional 26 questions were included to ask about barriers to OUD medications. This study attempted to answer the following research questions:

- What do probation department leaders perceive as barriers to probation officers’ linking and client access to OUD medications (MAT programs)?
- What do probation department leaders perceive is the extent to which themselves and their probation staff have been trained on, or have knowledge of, OUD medications?

This study was approved by the Illinois Criminal Justice Information Authority’s Institutional Review Board on February 1, 2018. The survey was created using Qualtrics software and emailed to 102 Illinois probation department directors in June 2018 and was closed in August 2018.

A total of 31 responses were received; five survey responses were excluded because more than one individual responded for their department (one survey per department was requested). The five respondents were eliminated based on 1) whether the respondent was the chief or director of probation (preferred respondent) or 2) randomly excluding those not submitted by the probation chief or director. The final sample size was 26 respondents (25% response rate) which represented 38 out of 102 Illinois counties (37%).^1^ Respondents were more frequently Probation Chiefs or Directors (69%). Responses were the opinions of those individuals who responded to the survey and may not accurately reflect individual staff opinions and knowledge. The 38 counties represented by the 26 probation department leaders were from all geographical locations in the state, as well as both urban and rural counties (Table 1). Further, the majority of responding probation department leaders identified opioid misuse as a *serious problem* in the county or counties they serve (77%, *n* = 20).

**Table 1:** Survey Participants (*N* = 26)

	n	% of sample	
Title			
Chief, Director Assistant Director	18	69.2%	
Coordinator, Supervisor, Manager	4	15.4%	
Probation Officer	3	11.5%	
Unknown/not specified	1	3.8%	
		% of 38 counties	% of 102 counties
Counties represented	38	100%	37.3%
Rural/Urban designation			
Urban counties	25	65.8%	24.5%
Rural counties	13	34.2%	12.7%
Region of the state			
Northern region counties	10	26.3%	9.8%
Central region counties	13	34.2%	12.7%
Southern region counties	15	39.5%	14.7%

## Results

### Barriers to use of OUD medications

Of the 25 probation department leaders who responded, 64% (*n* = 16) reported probation clients experienced at least a moderate degree of barriers to accessing medications—either 3 (*moderate), 4 (great extent), and* 5 (*very great extent). Seventy-three percent of the 22 responding probation department leaders agreed (4) or strongly agreed (5) that a barrier to accessing OUD medications included a l*ack of medical personnel experience (*n* = 16). One respondent commented on how doctor preferences guide medications used, and stated, “MDs tend to pick a medication and stick to it. Ours is not a big proponent of Vivitrol but uses Suboxone.” One-half of the sample agreed that cost (e.g. reimbursements, or concerns about costs for clients depending on insurance) was a barrier. One probation department leader noted that cost along with transportation/proximity to accessing medications are barriers, “Our small, rural community has no providers who offer MAT. I have had some drive to [nearest city] for it. The biggest barriers are cost and travel.” Forty-one percent noted a need for guidance on medications (*n* = 9), 36% indicated a lack of institutional knowledge (*n* = 8), and 23% reported regulations within their organization or jurisdiction may prohibit or constrain client use of medications (*n* = 5) (Table 2).

**Table 2:** Probation Department Leader Responses on Perceptions of Barriers to OUD Medications

		Agree/strongly agree	Neither agree/disagree	Disagree/strongly disagree
Barrier	N	%	%	%
Lack of access to medical personnel with expertise.	22	72.7	13.6	13.6
Agency has concerns about the cost of MAT/not enough funding.	22	50.0	18.2	31.8
Need for guidance.	22	40.9	36.4	22.7
Not enough institutional knowledge of medications and how they work.	22	36.4	22.7	40.9
Regulations prohibit use at the agency.	22	22.7	45.5	31.8
Agency has liability concerns (e.g. potential medication diversion/misuse).	22	18.2	45.5	36.4
Agency favors medicine-free treatment.	22	9.1	63.6	27.3
Clinical staff at your agency objects.	23	8.7	73.9	17.4
Administration in your agency objects.	23	4.3	87.0	8.7

### Training on, and knowledge of, OUD medications

One way to overcome some initial barriers is through education and awareness of OUD and MAT, typically through training that includes practical applicability of the information learned and how it relates to their job (Fixsen, Naoom, Blase, Friedman, & Wallace, 2005; Joyce & Showers, 2002; Smeele, Grol, Van Schayck, Van den Bosch, & Muris, 1999). Probation department leaders were asked about the extent to which they received training on each of the three FDA-approved OUD medications as well as their perception of the extent to which their staff has been trained on each of the three FDA-approved medications. Responses to these questions used a Likert scale, ranging from 1 (*no training at all*) to 5 (*a great deal of training*). Of the 25 probation department leaders, 84% reported their officers received *no* training or *a little* training on methadone (*n* = 21), 76% reported *no* training or *a little* training on buprenorphine (*n* = 19), and 64% reported *no* training or *a little* training on naltrexone (*n* = 16). Four percent of probation department leaders reported their officers received *a lot* to *a great deal* of training on methadone (*n* = 1), 4% on buprenorphine (*n* = 1), and 12% naltrexone (*n* = 3). Among the 26 probation department leaders, 65% indicated receiving *no* training or *a little* training on methadone (*n* = 17), 62% on buprenorphine (*n* = 16), and 46% on naltrexone. In addition, 12% of probation department leaders reported receiving *a lot* to *a great deal* of training on methadone (*n* = 3), 15% on buprenorphine (*n* = 4), and 15% on naltrexone (*n* = 4) (Table 3). Overall, this suggests that probation department leaders believed their staff—as well as themselves—have received limited training on each of the three OUD medications.

**Table 3:** Probation Department Leader Responses to, and Perceptions of Staff Training, Knowledge, Referrals, and Familiarity with OUD Medications (*N* = 26)

Amount of training	Not at all to a little		A moderate amount		A lot to a great deal	
	%	n	%	n	%	n
Probation department leader training						
Naltrexone	46.1	12	38.5	10	15.4	4
Buprenorphine	61.5	16	23.1	6	15.4	4
Methadone	65.4	17	23.1	6	11.5	3
Perceived staff training						
Naltrexone (*n* = 25)	64.0	16	24.0	6	12.0	3
Buprenorphine (*n* = 25)	76.0	19	20.0	5	4.0	1
Methadone (*n* = 25)	84.0	21	12.0	3	4.0	1
How knowledgeable are …	Not at all to slightly knowledgeable		Moderately knowledgeable		Very to extremely knowledgeable	
Probation department leaders about referrals for medications						
Naltrexone	26.9	7	34.6	9	38.5	10
Buprenorphine	34.6	9	26.9	7	38.5	10
Methadone	34.6	9	23.1	6	42.3	11
How open are/is …	Not at all to slightly open		Moderately open		Very to extremely open	
Leaders to client referrals for medications						
Naltrexone (*n* = 24)	4.2	1	8.3	2	87.5	21
Buprenorphine (*n* = 25)	8.0	2	20.0	5	72.0	18
Methadone (*n* = 25)	20.0	5	24.0	6	56.0	14
The agency to client referrals for medications						
Naltrexone (*n* = 24)	4.2	1	8.3	2	87.5	21
Buprenorphine (*n* = 25)	8.0	2	20.0	5	72.0	18
Methadone (*n* = 25)	20.0	5	24.0	6	56.0	14
How familiar are probation department leaders with …	Not at all to slightly familiar		Moderately familiar		Very to extremely familiar	
The use and purpose of medications						
Naltrexone	15.4	4	38.5	10	46.1	12
Buprenorphine	23.1	6	34.6	9	42.3	11
Methadone	15.4	4	50.0	13	34.6	9
The administration of medications						
Naltrexone	26.9	7	38.5	10	34.6	9
Buprenorphine	30.8	8	38.5	10	30.8	8
Methadone	19.2	5	53.8	14	26.9	7
How each of the medications work						
Naltrexone	34.6	9	23.1	6	42.3	11
Buprenorphine	34.6	9	26.9	7	38.5	10
Methadone	30.8	8	38.5	10	30.8	8
The effectiveness of medications						
Naltrexone	19.2	5	50.0	13	30.8	8
Buprenorphine	26.9	7	38.5	10	34.6	9
Methadone	19.2	5	50.0	13	30.8	8

The researchers also asked probation department leaders, *[h] ow knowledgeable are you about where to refer an eligible client to any of the following medication-assisted treatments?* Respondents were provided the three medication options to answer on a scale of 1 (*not knowledgeable at all*) to 5 (*extremely knowledgeable*). Forty-two percent reported they were *very* or *extremely* knowledgeable of where to refer clients for methadone (*n* = 11), 39% for buprenorphine (*n* = 10), and 39% for naltrexone (*n* = 10). Therefore, despite slightly less training or familiarity with methadone than the other medications, the number of respondents who knew where to refer someone for methadone was similar to the other medications. This may be due, in part, to the format in which methadone is obtained—only from a federally certified OTP.

### Familiarity with OUD medications for MAT

In addition, probation department leaders were asked about the extent to which they are familiar with each of the three FDA-approved medications in terms of their use and purpose, administration, how each works, and the efficacy of the medications. Responses to these questions included a Likert scale, ranging from 1 (*not at all familiar*) to 5 (*extremely familiar*). Most frequently, probation department leaders were *moderately familiar* with the use and purpose of methadone (50%, *n* = 13), *very* to *extremely familiar* with the use and purpose of buprenorphine (42%, *n* = 11), and *very* to *extremely familiar* with the use and purpose of naltrexone (46%, *n* = 12). Across the board, probation department leaders were *moderately familiar* with the administration of methadone (54%, *n* = 14), buprenorphine (39%, *n* = 10), and naltrexone (39%, *n* = 10). Most frequently, probation department leaders indicated they were *very* to *extremely familiar* with how naltrexone works (42%, *n* = 11), *very* to *extremely familiar* with how buprenorphine works (39%, *n* = 10), and *moderately familiar* with how methadone works (39%, *n* = 10). Conversely, 35% of probation department leaders were *not at all* or *slightly familiar* with how naltrexone works (*n* = 9), 35% for buprenorphine, and 31% for methadone. Probation department leaders most frequently reported they were *moderately familiar* with the efficacy of naltrexone (50%, *n* = 13), buprenorphine (39%, *n* = 10), and methadone (50%, *n* = 13). Overall, this suggest that while some probation department leaders indicate feeling familiar with these medications, more often than not, leaders only have little to moderate familiarity with the purpose, use, administration, efficacy, and mechanisms of OUD medications.

### Openness to referring clients to MAT

In addition, survey findings suggest probation department leaders perceived their staff to be generally more open to referring clients for treatment that include buprenorphine or naltrexone compared to methadone. Of the 23 probation department leaders who responded to questions regarding perceived staff openness to referring probationers to a buprenorphine provider, an Opioid Treatment Program (OTP) for methadone, or naltrexone, 72% (*n* = 18), 56% (*n* = 14), and 88% (*n* = 21), respectively, indicated they perceived their staff would be 4 (*very*) or 5 (*extremely*) open. The results were the same regarding probation department leaders being *very* or *extremely* open to referring clients to each of the three FDA-approved medications (Table 3).

## Discussion

The survey found probation departments experienced barriers for their clients to access medications for OUD that were consistent with prior studies (Farabee, 2018; Friedmann et al., 2012; Knudsen, Abraham, & Oser, 2011: Matusow et al., 2013). These barriers included lack of knowledge, lack of experience and training about MAT, high cost of medications, negative attitudes toward MAT medications, and restrictive department policies.

### Lack of knowledge, experience, and training

The survey found the most common barrier for probation client access to OUD medications was lack of experience by medical personnel. While there are no federal requirements to prescribe or provide naltrexone, the other two medications have more stringent regulations. Methadone can only be obtained from an OTP that is accredited and certified by SAMHSA, in addition to any state-required certification (Substance Abuse and Mental Health Services Administration, 2018). Buprenorphine requires doctors, nurse practitioners, and/or physician assistants training and a prescribing waiver from Drug Enforcement Administration, which limits the number of possible patients (Substance Abuse and Mental Health Services Administration, 2018). Currently, there is a national shortage of healthcare providers to prescribe buprenorphine (Ross Johnson, 2018). Healthcare providers may be against agonist treatment, lack time for more patients, or do not receive full insurance reimbursement (Huhn & Dunn, 2017). Prior surveys of physicians found an increased willingness to prescribe if provided information on local counseling resources, mentorship with an experienced provider, and more education on OUDs (Huhn & Dunn, 2017; Hutchinson, Catlin, Andrilla, Baldwin, & Rosenblatt, 2014; Walley et al., 2008). It is only recently that some medical schools have incorporated a specialization—or even courses—in addiction medicine to their curriculums (Wood, Samet, & Volkow, 2013). Additionally, one other way to increase client access to medical care can be through the use of, or client linkage to, telehealth programs—programs that connect individuals to medical services, including screenings, counseling, and medication through telephone-, internet-, video-, or smartphone application-based services (National Association of State Alcohol and Drug Abuse Directors, Inc. [NASADAD], 2009). This is particularly helpful for those in rural areas where providers may be scarce, as well as individuals who face other barriers to accessing treatment—child care, transportation, and work schedules (National Association of State Alcohol and Drug Abuse Directors, Inc. [NASADAD], 2009).

Probation departments also had deficiencies in knowledge—19% to 23% received no prior training on the OUD medications. In addition, two of the barriers cited by probation departments—need for guidance and lack of institutional knowledge—support their need for education, consistent with prior studies (Knudsen et al., 2011). Training may be an effective way to inform probation officers on OUD and MAT, helping to increase referrals to MAT or healthcare providers who may be able to guide their client towards the right MAT program. In a study of a multi-site training for probation, Medication-Assisted Treatment Implementation in Community Correctional Environments (MATICCE) found training and planning between probation and treatment providers to resolve barriers was effective in changing attitudes and intent to refer clients to MAT (Ducharme et al., 2013; Friedmann et al., 2015). Recent work by McCarty, Rieckmann, Green, Gallon, and Knudsen (2004) found counselors had more positive attitudes toward buprenorphine following training.

### Cost of medications

Half of the surveyed probation departments cited cost as a barrier to medications. Probation departments can work with clients and healthcare providers to determine costs and explore payment options including help accessing public assistance or personal insurance (Substance Abuse and Mental Health Services Administration, 2018). Methadone and buprenorphine have cheaper generic formulations; injectable naloxone (brand name Vivitrol) does not. There are policy obstacles of private and public insurance that need to be addressed, including limitations on dosage or length of treatment, as well as initial authorization and reauthorization requirements (Volkow, Frieden, Hyde, & Cha, 2014). For example, the American Medical Association (AMA) recommends the federal government suspend waiver requirements for physicians prescribing buprenorphine under Medicaid and Medicare (American Medical Association, 2018). In addition, medical providers have called for decreasing barriers to client access to these medications, such as eliminating prior authorizations for these medications—though healthcare providers have been slower to incorporate this fully into practice (Clark et al., 2014). In addition, there are other costs including transportation back and forth between visits, childcare, and time spent at office appointments (Jones et al., 2009).

### Attitudes towards medications

Several barriers centered on agency and individual attitudes, including clinical and administration staff objections and agency preferences for medicine-free treatment. Some probation departments have been hostile towards and prohibit probationers from taking medications, believing it is “trading one drug for another” (Hora, 2014: National Institute on Drug Abuse, 2018; Sharfstein, 2018). The medications used as part of MAT do not produce a “rush,” but offer an extended stabilization with reduced opioid cravings (National Institute on Drug Abuse, 2018). Despite the strong evidence-base, partial and full agonist medication for OUD treatment is at odds with the predominant philosophies grounded in abstinence-based and 12-step therapies (Knudsen et al., 2011). OUD treatment decisions should be made between a medical provider and the patient, integrated into primary care, and given parity with treatment of any medical disorder (Farabee, 2018).

### Department policies

Probation officers may not have the autonomy to refer or allow their clients to use medications for OUD—nor are they medical prescribers or provides—and as such, they must follow their agency’s policies and procedures, in addition to court orders (Mitchell et al., 2016). In this study, 32% of survey respondents noted that regulations prohibited use. Departments may have concerns about liability of misuse or “diversion” or illegally sharing prescribed medications with others (Farabee, 2018). The National Association of Drug Court Professionals (NADCP, 2011) resolution guides drug courts that they “do not impose blanket prohibitions against the use of MAT for their participants” (p.2). In fact, federal laws prohibit discrimination against, and offers protection for, those receiving medications for OUD (Nordstrom & Marlowe, 2016), though currently, nothing precludes probation departments from providing access to MAT services. Increased access to these medications necessitates policy changes at the agency, state, and federal level for providers to prescribe, clients to access, and practitioners to better understand the use and efficacy of these medications and ways to refer clients for medical assessment and treatment.

### Limitations and future research

The findings of this study should be viewed with caution due to some limitations. A main study limitation was the low sample size which is likely not representative of the entire state’s probation departments nor probation departments across the U.S., limiting the generalizability of the findings. Despite variability of respondents’ geographical representation of urban and rural counties and locations in the state, there may have been selection bias. It may be that probation department leaders who may already be aware of MAT and medications for OUDs were more likely to respond. Further, based on the number of probation department leaders’ responses to the seriousness of their jurisdiction’s opioid misuse problem (77% reported it as a serious problem), the sample is likely biased towards jurisdictions with more serious opioid misuse problems compared to those who may have more moderate or slight opioid misuse problems. Second, responses were from probation department administrators and directors and not a reflection their staffs’ opinions, knowledge, and practices. This limits the ability to understand how staff, who provide these referrals and linkages, may view or understand OUD medications and SUDs. For example, Friedmann et al. (2012) noted that much of a criminal justice practitioner’s views on MAT are predicated on their understanding of addiction and SUDs more generally. Third, some respondents did not answer all survey questions, further limiting the generalizability of some questions.

Future research could employ sampling and recruitment methods that may yield a larger and more representative sample size, such as stratified random sampling. This may help reduce sample bias and increase sample representativeness. In addition, future research could directly survey probation officers and staff about their knowledge, opinions, and practices as these individuals provide the direct linkage and referrals to MAT and other supportive services. This can also help parse out whether there is a difference or similarity in probation department leaders and staff regarding barriers, knowledge, and understanding of how MAT for OUDs can be an effective treatment option—one which should be determined by a medical provider and not the courts or corrections. Future research should examine the impact of training (including training models, delivery, and length) on probation officer attitudes and subsequent decisions and actions. While this survey did not reveal differences in barriers in rural and urban areas, further research should explore this more in-depth as there may be more barriers to medications in areas with less accessibility to healthcare and SUD treatment providers who prescribe medications and/or OTPs.

## Conclusion

For this study, probation department leaders were surveyed, resulting in identification of several barriers they perceive in linking probation clients to medications for OUD, consistent with prior research. Barriers included lack of knowledge of medical personnel and probation departments; cost; department policies; and negative attitudes towards medications. These findings indicate a need for administration and staff training about SUDs, and specifically OUDs; MAT may interface with probation and what a probation officer needs to know when it comes to MAT and compliance; interagency collaboration between probation department staff, healthcare providers, and SUD treatment providers; and state, county, and departmental policy changes to increase access to, and use of, medications for probation clients with OUD.

As stated by Ducharme et al. (2013),

Needed are evidence-based strategies for changing the business practices of organizations and systems to fully integrate drug abuse treatment services for drug-involved individuals under criminal justice supervision, in a manner that respects and supports dual goals of public health and public safety (p.6).

While probation officers are tasked with monitoring their clients in the interest of public safety, they are also called upon to help clients with behavior change (Lovins et al., 2018). Helping clients get the individualized treatment they need for OUD, in conjunction with other necessary social and human services, is part of the dual role of a probation officer. Research strongly supports that the use of prescribed medications for the treatment of OUD will allow probation clients to be more stabilized, increase engagement in behavioral therapies, better adhere to other probation requirements, and have improved outcomes including reduced recidivism and mortality.

## Abbreviations

AMA: American Medical Association
CJ-DATS: Criminal Justice-Drug Abuse Treatment Studies
FDA: U.S. Food and Drug Administration
MAT: Medication-Assisted Treatment
MATICCE: Medication Assisted Treatment Implementation in Community Correctional Environments
NA: Narcotics Anonymous
NADCP: National Association of Drug Court Professionals
NCJTP: National Criminal Justice Treatment Program
OTP: Opioid Treatment Program
OUD: Opioid Use Disorder
SAMHSA: Substance Abuse and Mental Health Services Administration
SUD: Substance Use Disorder

## Footnotes

1 Some probation departments serve more than one county.
